# Microbiome Modulators and Mood Disorders: Using a Multi-Strain Probiotic - Bio-Kult® Advanced - in Patients With Low Mood

**DOI:** 10.1192/bjo.2022.255

**Published:** 2022-06-20

**Authors:** Deepti Aswani, Richard Day, Vineetha Vijayakumar, Malwina Naghibi, Nicola Wolstenholme, Grace Barker

**Affiliations:** ADM Protexin, Somerset, United Kingdom

## Abstract

**Aims:**

The aim of this proof-of-concept study was to understand the effect of daily intake of a 14-strain probiotic on mood, reward learning and emotional and cognitive processing in adults with low mood in the absence of prescribed medication. Salivary cortisol was measured as a marker for physiological stress.

**Methods:**

In this parallel-group double-blind, placebo-controlled trial, 80 healthy adults with self-identified low mood were randomised to receive either the 14-strain probiotic or placebo for a duration of 4 weeks. Data were collected from participants at baseline (week 0) and post-intervention (week 4).

**Results:**

Probiotic intake significantly reduced depression scores (by 50%) compared to baseline, as measured by the Patient Health Questionnaire-9 (PHQ-9) scale (p < 0.05). Analysis of individual items in the PHQ-9 revealed that participants taking probiotics reported improved concentration relative to baseline (+ 51%, p < 0.05) and felt less tired compared to placebo (−21%, p < 0.01).

Regarding emotional processing, the probiotic group was more accurate at recognising facial expressions compared to those receiving placebo (facial emotion recognition test, +12%, p < 0.05). Furthermore, the probiotic group performed less well at the reward learning task relative to the placebo group (probabilistic instrumental learning task, p < 0.05) and was less vigilant to emotional cues compared neutral cues (dot-probe unmasked test, −8%, P < 0.05). The probiotic group also showed increased susceptibility to emotional interference during a cognitive learning task, relative to placebo (auditory visual learning task, −18% p < 0.05).

The study also revealed a downward trend in salivary cortisol in the probiotic group over 4 weeks.

Together, these results suggest that probiotics may work via a different psychological mechanism to that of conventional antidepressants. In other words, probiotics may work by reducing emotional salience across all emotions whereas conventional antidepressants are thought to work by increasing bias to positive emotional cues and decreasing bias to negative ones.

**Conclusion:**

These data suggest that intake of Bio-Kult® Advanced has an effect on mood and that this is achieved in ways distinct from the effects of pharmacological antidepressants. While more research is needed, these results suggest that certain probiotics could form part of an ‘early intervention’ strategy for people experiencing low mood. A second randomised controlled trial (currently recruiting) will provide data on this intervention in patients with a formal diagnosis of depression undergoing concurrent pharmacological treatment.

ClinicalTrials.gov Identifier: NCT03801655

